# The Early Days of NK Cells: An Example of How a Phenomenon Led to Detection of a Novel Immune Receptor System – Lessons from a Rat Model

**DOI:** 10.3389/fimmu.2014.00283

**Published:** 2014-06-16

**Authors:** Bent Rolstad

**Affiliations:** ^1^Immunobiological Laboratory, Department of Anatomy, Institute of Basic Medical Sciences, University of Oslo, Oslo, Norway

**Keywords:** Ly49 receptors, MHC-I molecules, Rat NK alloreactivity, activating receptors, NK receptor repertoire, inhibitory receptors, natural killer cells, rat models

## Abstract

In this review, I summarize some of the early research on NK cell biology and function that led to the discovery of a totally new receptor system for polymorphic MHC class I molecules. That NK cells both could recognize and kill tumor cells but also normal hematopoietic cells through expression of MHC class I molecules found a unifying explanation in the “missing self” hypothesis. This initiated a whole new area of leukocyte receptor research. The common underlying mechanism was that NK cells expressed receptors that were inhibited by recognition of unmodified “self” MHC-I molecules. This could explain both the killing of tumor cells with poor expression of MHC-I molecules and hybrid resistance, i.e., that F1 hybrid mice sometimes could reject parental bone marrow cells. However, a contrasting phenomenon termed allogeneic lymphocyte cytotoxicity in rats gave strong evidence that some of these receptors were activated rather than inhibited by recognition of polymorphic MHC-I. This was soon followed by molecular identification of both inhibitory and stimulatory Ly49 receptors in mice and rats and killer cell immunoglobulin-like receptors in humans that could be either inhibited or activated when recognizing their cognate MHC-I ligand. Since most of these receptors now have been molecularly characterized, their ligands and the intracellular pathways leading to activation or inhibition identified, we still lack a more complete understanding of how the repertoire of activating and inhibitory receptors is formed and how interactions between these receptors for MHC-I molecules on a single NK cell are integrated to generate a productive immune response. Although several NK receptor systems have been characterized that recognize MHC-I or MHC-like molecules, I here concentrate on the repertoires of NK receptors encoded by the natural killer cell gene complex and designed to recognize polymorphic MHC-I molecules in rodents, i.e., Ly49 (KLRA) receptors.

## Introduction

**Figure 1:**
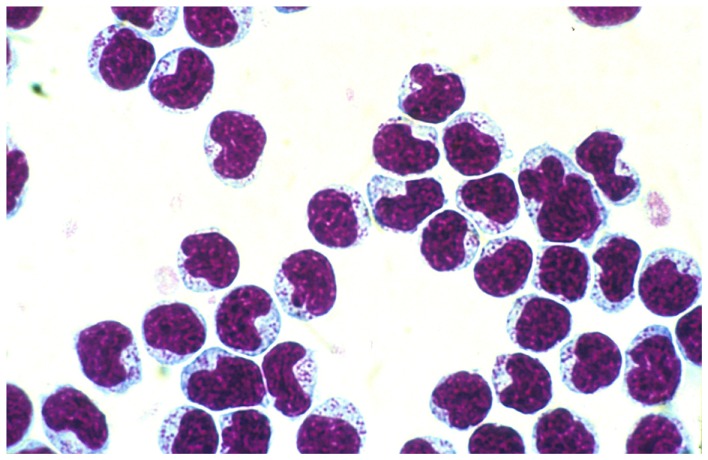
**Natural killer cells separated from peripheral blood of F344 rats on a Percoll gradient**. Note the large azurophilic granule in the cytoplasm.

Natural killer (NK) cells were originally defined as lymphocytes with spontaneous reactivity against certain tumor cells. The basis for recognition and the receptor repertoire they employed were unknown. The nomenclature was debated, but eventually settled by Eva Klein and coworkers as NK cells in the mid 1970s (1, 2). Ronald Herberman and coworkers identified them morphologically as large granular lymphocytes (LGL) (3, 4) (Figure 1). Whether these cells were lymphocytes with unique receptors for cancer cells or their recognition mechanisms were just byproducts of unspecific T cell recognition of target cells, was a matter of debate. At that time, the molecular structure of the TCR and their cognate MHC ligands were not known.

## Mouse NK Cells Recognize Polymorphic MHC-I Ligands via a Repertoire of Inhibitory Receptors

A contrasting phenomenon also ascribed to NK cells was “hybrid resistance” described initially by Cudkowitz and Bennett (5–7), i.e., irradiated F1 hybrid mice between two inbred “parental” mouse strains often failed to accept parental bone marrow cell (BMC) transplants. Certain MHC products, more specifically MHC-I molecules, were clearly involved (8). However, this form of “rejection” defied the classical laws of transplantation: F1 hybrid mice between mice strains differing at the MHC and expressing all the MHC-I molecules from both parental strains were still able to “reject” parental BMC. The accumulating body of *in vivo* experiments indicated unequivocally that this kind of rejection was mediated by NK cells (9). It became so strong that it stimulated researchers to revise the notion that the immune system was mainly designed to recognize “foreign” or diseased “self” molecules. Klas Kärre and Hans-Gustav Ljunggren introduced the ingenious “missing self” concept of NK recognition (10–12), which *prima facie* gave a unifying explanation for both hybrid resistance and the rejection of tumor cells that had low or absent expression of their MHC-I molecules. These and later studies rested on the assumption that NK cells possess a repertoire of inhibitory receptors expressed on different subpopulations of NK cells and that these receptors recognize unmodified “self” MHC-I. Hybrid resistance could then be explained by the assumption that some NK cells failed to express inhibitory receptors for MHC-I molecules from one or the other of the parental strain BMC and therefore killed them through “missing self.” NK cells can be activated via a repertoire of receptors for other ligands present on both normal and neoplastic cells, especially, the NKG2D receptor being present on all NK cells and recognizing stress induced ligands on target cells (13). However, the presence of sufficient amounts of unmodified “self” MHC-I on the cell surface inhibited the NK cell subset with inhibitory receptors for such ligands from killing (14).

The missing self-hypothesis has over the decades since its conception been substantiated by the detection of a steadily increasing repertoire of inhibitory NK receptors that can recognize “self” MHC molecules and also inhibit other activating receptors present on the same NK cell. Although the complex interplay between activating and inhibitory receptors has led to several modifications of the “missing self” hypothesis, the basic concept that NK cell activation depends on stimulation of activating receptors overriding inhibitory receptors has emerged from the original concept.

## Rat NK Cell Alloreactivity: A Model Organism for Studying Some Unorthodox Patterns of NK Cell Allorecognition

The missing self-hypothesis in its simplest form was challenged by a phenomenon first observed in the rat: allogeneic small non-dividing and recirculating lymphocytes, when injected i.v. into naïve normal recipients, were sometimes eliminated as soon as they had left the blood stream (15, 16). MHC genes were clearly involved (17) but other factors also played a role, since this rapid elimination was also dependent on genes outside the MHC complex (15, 18, 19). The elimination took place in the lymphoid tissue (15, 16) more specifically within the T cell areas of lymph nodes and spleen (20). However, T cells by themselves were not involved in this acute rejection (see below). This phenomenon, described extensively in a volume of Immunological Reviews (Elimination of Allogneic lymphoid cells vol. 73 1983) was termed *natural cytotoxicity* by Barbara Heslop (19) or allogeneic lymphocyte cytotoxicity (ALC) by us (21), emerged from a series of experiments stimulated by my supervisor William L. Ford at the Dunn School of pathology in Oxford already in the mid 1970s (15, 22). The intention was to study the role of MHC compatibility in controlling lymphocyte recirculation. Since the molecular basis for MHC restriction was not known at the time, some researchers believed that this restriction might apply also to interactions between lymphocytes and non-hematopoietic cells. This led further to the idea that the interaction between small recirculating lymphocytes and the high endothelial venules (HEV) of the lymphatic tissues, where recirculating lymphocytes emigrate from the blood into the lymphoid tissues, was dependent on MHC compatibility between the lymphocytes and the HEV (23, 24). In the spirit of the Gowans/Ford pioneering studies on lymphocyte recirculation between blood and lymph (25–27) I conducted a series of experiments where recirculation of allogeneic lymphocytes was compared with that of syngeneic lymphocytes in immunologically naïve animals. I used three different rat strains, all differing at the MHC: PVG, AO, and DA rats. With appropriate dual radioactive labeling of the cells the recirculation of allogeneic and syngeneic lymphocytes could be monitored in the same recipient (15).

What clearly emerged from these studies was that i.v. injected recirculating lymphocytes entered the lymphoid tissue from the blood equally well regardless of whether they were MHC compatible or incompatible with the host, i.e., lymphocyte–HEV interactions were not MHC restricted (15). Later autoradiographic studies substantiated this conclusion (20). However, i.v. injected allogeneic lymphocytes completely failed to enter the efferent lymphatic vessels, i.e., the thoracic duct (15), which normally occurs between 12 and 24 h after i.v. injection of lymphocytes syngeneic with the recipient. Presumably, they had been eliminated within the lymphatic tissues before returning via the main lymphatic vessels to the blood. An *in vivo* cytotoxic assay was designed to test this. After injection of ^51^Cr labeled allogeneic lymphocytes into rats with an indwelling thoracic duct cannula, they completely failed to reappear in thoracic duct lymph, but the radioactivity was instead found in the lymph fluid (28, 29). This assay also allowed us to study the kinetics of elimination. By monitoring the radioactivity among recirculating lymphocytes and in lymph fluid in timed collections from the thoracic duct, we observed evidence of incipient elimination already within the first 15–30 min after i.v. injection, i.e., as soon as the lymphocytes had penetrated HEV and entered the lymphatic tissues, and it was by and large complete by 24 h (28, 29). Further autoradiographic studies visualized the site of allogeneic lymphocyte destruction: within the paracortex of lymph nodes and the PALS of the spleen (20). Since both T and NK cells are present in these locations, we could not exclude a contribution of T cells. However, the genetics of ALC and that of T cell alloreactivity were completely different as alluded to below.

In my first publication (15), I saw some general traits of ALC and hybrid resistance in mice that led me to postulate already in 1979 that both phenomena might be a manifestation of a common underlying mechanism assigned to the function of NK cells. However, there were some notable differences:

- In contrast to hybrid resistance, MHC homozygous inbred rats sometimes rejected F1 hybrid lymphocytes: e.g., PVG rats rejected lymphocytes from (PVG × DA)F1 or (PVG × AO)F1 rats almost as well as fully allogeneic DA or AO cells (21, 28). So the presence of one MHC haplotype identical between the donor and recipient resulting in exposure of “self” MHC molecules on donor cells to the recipient did not guarantee survival of the injected cells.
- The ALC-mediated rejection was often asymmetric, e.g., PVG rats rejected DA lymphocytes while there was no rejection in the other direction (15). Whatever receptor systems were employed, they diverged highly from the TCR recognition of allogeneic MHC-I, where strong recognition by CTL in both directions had been demonstrated.
- In contrast to tumor recognition and hybrid resistance, this form of rejection was against small non-dividing recirculating lymphocytes, indicating that NK cells recognized antigens on highly differentiated cells and not only tumor cells or multipotent hematopoietic precursors.
- In contrast to T cell alloreactivity, which is mainly directed against molecules encoded by the classical MHC-I region termed *RT1-A* in the rat, ALC was also controlled elements within the non-classical class I region termed the *RT1-CEM* region (17) (see below).
- Cold target competition studies defined several NK “allospecificities” determined by the MHC (30–32). Some of the antigens were present in the area of the rat MHC that does not restrict T cell responses to conventional antigens, i.e., in the *RT1-CEM* region (32–34) (see below). Further experiments identified several polymorphic genes in this region that activated NK cells. This assigned a novel function to this poorly investigated string of MHC-I genes (35, 36). These MHC-I molecules have about a 10-fold lower expression at the cell surface than classical MHC-I and had then been disregarded as having functional importance in classical immune recognition (37).

The strongest evidence that ALC was mediated by NK cells was obtained from studies in athymic nude rats:

- They are devoid of alloreactive T cells but have increased numbers of alloreactive NK cells and eliminated the allogeneic lymphocytes via the ALC mechanism more strongly than normal rats (28).
- Removal of NK cells from these rats with specific antibodies led to a near complete extinction of ALC (29).
- Removal of B cells and immunoglobulins did not affect ALC thus ruling out the contribution of alloantibodies (29).
- Unlike adaptive immune responses but similar to NK alloreactivity (NKa) the ALC mechanism did not develop until after the fourth week of life (29).

My supervisor at the time, William L. Ford strongly urged me to continue these studies. His ability to look into future was supported by his statement: “this phenomenon will be of the greatest biological significance.” Alas, he did not live long enough to see that his predictions came true.

## The Rat MHC (*RT1*): Genetic Organization and Role in NK Alloreactivity

**Figure 2:**
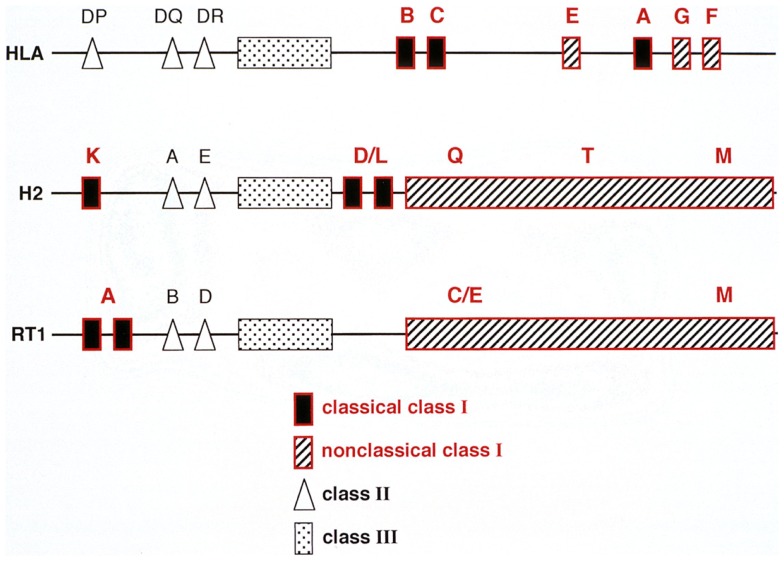
**A comparison between the genetic organization of the human (HLA), mouse (H2), and rat (RT1) MHC complex**. Note that in all three species genes encoding class I, II, and III molecules are present. However, their positioning within the MHC are different: the humans lack a centromeric MHC-I encoding region whereas rats (RT1) lack mouse D/L homologs telomeric to the class II but instead has a string of genes in the centromeric part of the RT1.CEM region termed RT1.CE, some of which are expressed, can present peptides and are also recognized by NK cells.

In order to understand mechanisms underlying ALC and its relationship to hybrid resistance, a brief description of the rat MHC (*RT1*) is warranted. The *RT1* shows several similarities with both the mouse and human MHC in that it contains regions encoding both MHC-I and II molecules and centrally a class III region with genes encoding members of the TNF, HSP, and some complement factors (33) (Figure 2). The presence of highly conserved non-polymorphic framework markers like KE3, Bat 1, and MOG at certain checkpoints within the MHC in these three species (33) further indicates that the mouse, rat, and human MHC have developed from a common ancestor. The rat and mouse MHC resemble each other in that MHC-I genes are flanking the class II and III region on each side. However, unlike the mouse, the rat classical MHC-I molecules restricting T cells to environmental antigens are located mainly on the centromeric side of the complex (33). Here, dependent on the rat strain, one, two, or three classical MHC-I loci are present, each restricting individual T cell responses (38). Telomeric to the class III region is a string of non-classical class I genes where the most centromeric part has replaced the H2 D/L in the mouse. I will concentrate on this region termed *RT1-C/E*, since here the polymorphic MHC-I genes relevant for NK allorecognition are present (32) (Figure 2). Again, there is a great variety of the number of MHC-I genes present in the *RT1-C/E* region between rat strains. In BN rats several genes are present, some are pseudogenes but most are full length genes, expressible on the cell surface and some of them can also present peptides (39). However, their expression at the cell surface is about 10 fold lower than the classical *RT1-A* encoded molecules (40).

## *In vitro* Assays for NK Alloreactivity: The NK Gene Complex

The final proof that NK cells were alloreactive and could be responsible for ALC came with our device of an *in vitro* test for NK cell alloreactivity: purified NK cells killed MHC-incompatible lymphocytes in a standard 4 h *in vitro* cytotoxic assay (41), and they specifically recognized gene products encoded by the MHC-I regions (31, 32). Recognition furthermore showed the same patterns as those observed in our ALC experiments, i.e., both classical (*RT1-A*) and non-classical (*RT1-CE*) MHC-I molecules were targeted (32, 34, 42). However, *RT1-A* encoded molecules mainly inhibited ALC and NKa *in vitro* while *RT1-CE* encoded molecules both could either inhibit or stimulate alloreactivity depending on the rat strain combinations used. Thus, a putative specialization of classical and non-classical MHC-I regions in control of NKa in the rat was clearly different from the mouse. Furthermore, a novel function was assigned to this *RT1-CE* region.

This initiated a search for receptors on NK cells that could mediate ALC *in vitro*. Moretta and coworkers had already identified receptors on human NK cells termed killer cell immunoglobulin-like receptors (KIR), that upon interaction with MHC-I molecules inhibited NK reactivity (43, 44). Later KIR receptors activated by HLA were also identified (45). Karlhofer and Yokoyama identified a receptor termed Ly49A on mouse NK cells and belonging to the family of lectin-like receptors encoded by the natural killer cell gene complex (NKC) (46). This region contains several families of lectin-like receptors belonging to the type two membrane proteins with the carboxy terminus extracellularly. It contains receptors that recognize a multitude of ligands most of which are MHC or MHC-like. I will in the following concentrate on the Ly49 receptor family, which is most relevant for understanding the alloreactivity of NK cells. The interaction between Ly49A and H2D^d^ inhibited NK cell from killing the target (47, 48). The Ly49 family of the NKC was also present in the rat (49) and this led to a search for Ly49 members that activated rather than inhibited ALC. The identification of an NKa locus in the rat dominantly controlling ALC gave additional strong circumstantial evidence for the presence of activating members of the Ly49 family (49), but we had at the time no molecular evidence for this.

## Identification of Both Stimulatory and Inhibitory MHC-I Recognizing Ly49 Receptors in the Rat

Ironically, the first rat Ly49 member that we molecularly cloned and functionally characterized was an inhibitory one with an immuno tyrosine inhibitory motif (ITIM) in its cytoplasmic domain, indicating inhibition. Its recognition of rat MHC-I molecules was highly specific in that only one of the two classical class I molecules present in the PVG rat strain, RT1-A1^c^, was recognized by the receptor and we called it Ly49i2 (50, 51). Our initial failure to identify activating Ly49 receptors in the rat was due to the fact that the cells that we used as transfectants for expression cloning lacked the adaptor protein DAP12 needed to stimulate activating downstream pathways for these receptors in NK cells.

However, with the access to the technology needed for coexpression of DAP12 and the candidate activating receptor genes, we eventually identified the first series of activating rat Ly49 receptors with the mAbs DAR13 or STOK6. One of these receptors, Ly49s3, was clearly an activating receptor for a non-classical MHC-I RT1-CE antigen (52, 53). Further research along these lines identified a repertoire of both activating and inhibitory Ly49 receptors, differentially expressed on subpopulations of NK cells (53, 54). This gave the Ly49 region a central place in rat NKa as it did in mice.

Being beaten time wise by researchers identifying activating Ly49 receptors for MHC-I molecules in the mouse (55, 56), is rat NK cell alloreactivity still worth pursuing or do we simply study “big mice”? We believe no. Although mice and rats have an overall superficial similarity in the construction of their NKC with receptors for both MHC-I and other molecules, the repertoire of activating rat Ly49 receptors in rats by far outnumbers that in the mouse (57). Also, the different construction of the MHC between rats and mice (Figure 2) indicates that the 10–20 million years that have separated mice and rats from a common ancestor has had profound effects on the tuning of this part of the innate immune system. Functionally, rat NK receptors may also have adapted differently to the microbial flora the two species have been exposed to. In mice lacking the adaptor molecule DAP12 associated with activating Ly49 NK receptors, the innate immune response to the intracellular bacterium *Listeria monocytogenes* (LM) is enhanced (58). In contrast, NK cells have a primary role in early resistance to LM in the rat (59, 60). Removal of NK cells from the recipient abrogates the early resistance against LM and athymic nude rats with increased numbers of alloreactive NK cells eliminate LM more efficiently than normal rats (59). Again non-classical MHC-I molecules play a central role, since infection of target cells with LM led to increased expression of a non-classical MHC-I antigen and this stimulated an activating NK cell receptor (60). It would be informative to compare complete maps of next generation sequences (NGS) of the whole NKC in several mouse and rat strains to pinpoint the regions and mechanisms leading to this divergence.

## Inhibitory versus Activating Receptors, What Came First, the Hen or the Egg?

In the species where NK receptor repertoires for polymorphic MHC-I molecules are studied most extensively, there is a striking pairwise appearance of several of these receptors: some have almost identical amino acid sequences in their extracellular antigen binding domains but different signaling properties (61). An intracellular ITIM motif indicates that it is an inhibitory receptor, while the presence of a charged amino acid in the transmembrane region (Arginine or Lysine) points to an activating function since it can bind activating adaptor molecules such as DAP 12. This suggests that the antigen binding extracellular part of such receptor pairs originates from a common gene. The great resemblance in both the gene and amino acid sequences of the receptor site indicates that subtle mechanisms may account for this diversification. The prevailing one is that activating receptors arose from inhibitory ones under pressure by pathogens producing decoy MHC-like ligands that engaged the inhibitory ones and thus shut down NK cell recognition though missing self (62, 63). Some of these activating receptors can recognize such decoy viral peptides directly such as the MCMV m157 protein recognized by the Ly49H receptor thus making Ly49H receptor positive mice more resistant to infection (64). Alternatively, activating receptors may recognize MHC-I molecules directly, but the peptides loaded onto the MHC-I may be critical for its avidity to the receptor. Certain viral peptides presented by MHC-I may increase the avidity for binding to activating KIR (65).

The mechanisms creating the minute differences between inhibitory and corresponding activating receptors are speculative, but gene conversion, gene duplications, and deletions may contribute. One mechanism contributing to this close relationship is “gene homogenization” described by Fossum and coworkers. Here, genes encoding the extracellular portion of the receptors are tightly connected, highly similar or identical, and forced to be expressed either as activating or inhibitory depending on coupling to genes encoding the inhibitory or activating transmembrane or intracellular motifs (66). Gene conversion may be a central mechanism here. The mechanisms leading to homogenization are not known.

Not only at the genetic but also at the functional level the similarity between such inhibitory and activating receptor pairs is striking so that such receptor pairs can recognize the same ligand. E.g., Ly49A is inhibitory while Ly49D is activating but both bind to H2D^d^ in the mouse (48, 67). KIR2DL1 and KIR 2DS1 both recognize HLA-C2 in humans (68, 69), and the Ly49s5/49i5 pair can recognize similar non-classical (RT1-CE) MHC ligands in the rat (54). In mice and humans, sometimes these paired receptors are expressed in the same NK cells, which may create a problem in understanding how receptors with identical or similar ligands but with opposing functions may create a productive immune response. Our present incomplete knowledge about which mechanisms control the differential expression of these receptor systems in various NK cell subsets and also how the minute differences in amino acid sequences in the ligand binding site of such receptors may affect their avidity and binding site for their cognate MHC-I ligands are items that need to be investigated.

The other issue is that, unlike T cells, each NK cell has a unique repertoire of both activating and inhibitory receptors, many of which recognize MHC-I molecules, and this composition varies between different NK cells. One example is the Ly49 i5 and s5 receptor pair in the rat. They are both expressed in NK cells in PVG rats, can bind avidly to MHC-I ligands encoded in the *RT1-CE* region but are present on different subpopulations of NK cells and will therefore not cancel each other out (54). A most important mechanism for control of the activating NK receptors is that they may be present, but hyporesponsive unless the NK cell at the same time expresses enough inhibitory “self” receptors to keep the activating receptors in check, a phenomenon termed “licensing” or “education” (70). Only when an activating receptor recognizes an MHC ligand altered by disease and possibly with higher affinity than its “healthy” counterpart, could activation override inhibition. This is an elegant mechanism to avoid autoreactivity of the activating Ly49 receptors. Here are several challenges for further research. One is to get a more complete picture of all the Ly49 receptors that NK cells in different mouse or rat strains that recognize MHC-I ligands. Furthermore, how does a pathogen affect the expression of MHC-I molecules on an infected target that may allow NK cell activation to dominate over inhibition? The diversity of the microbiome of importance for the generation and maintenance of the innate immune system is far from completely investigated. Microbial interactions with target cells may alter their expression of MHC-I and therefore also affect recognition by activating and inhibitory receptors on NK cells differentially.

Since we do not yet have a complete picture of all the cellular and molecular events leading to NK cell repertoire formation for polymorphic MHC-I molecules, the question asked in the heading of this section may be more of a philosophical nature than one with practical implications, since such activating/inhibitory pairs of receptors with similar or identical ligand binding sites are widely distributed throughout the innate immune system (63). Three possibilities may be considered: (1) activating receptors evolved from the inhibitory ones under pressure from pathogens as alluded to above (62) (2) coevolution of activating and inhibitory receptors, i.e., coupling of the MHC-I binding site of a receptor to inhibitory or activating motifs could have occurred simultaneously, e.g., though homogenization (66). (3) However, whenever there is formation of an NK receptor repertoire designed to detect a multitude of foreign invaders, e.g., bacteria or viruses infecting host cells or otherwise “unhealthy” cells, I presume that activation will be a primary driving force (35, 66). With increased knowledge of the evolution of the receptor systems, the control of their expression in different NK cell subpopulations and how, e.g., microbial forces have shaped these repertoires in different species, we may get a more precise view of how this important arm of the innate immune system was created and finely tuned to protect us from disease.

## Back to the Rat, What Additional Information Can This Species Give Us about the Function and Regulation of NK Receptors for Polymorphic MHC-I Molecules?

Given the repertoire of activating Ly49 receptors in the rat and the polymorphic non-classical MHC-I (RT1-CE) ligands they recognize, I find it unlikely that such a diverse recognition system has arisen from inhibitory Ly49 receptors alone. How, then, can education of such a system be reconciled with a control by inhibitory Ly49 receptors recognizing classical MHC-I, i.e., licensing? The fact that activating Ly49 receptors are functional on mature rat NK cells and need no extra education to immediately recognize non-classical MHC-I of a different haplotype (54) puts into doubt that licensing is the only mechanism here. Another possibility is that non-classical MHC-I (RT1-CE) molecules in the rat being expressed at a much lower level than the classical RT1-A molecules (40) leading to so weak recognition by activating Ly49 receptors that it escapes significant NK cell stimulation and also control by the inhibitory receptors. Only after infection of a target cell with, e.g., with *Listeria* will the RT1-CE ligand be upregulated to the threshold needed for stimulation (60). Alternatively, infection may alter the conformation of RT1-CE molecules so that binding to and activation of Ly49 receptors is enhanced. Nonetheless, the discovery of a repertoire of activating Ly49 receptors for non-classical MHC-I molecules in the rat poses a new dimension to how the Ly49 repertoire has evolved. One consequence of such repertoire formation, where subgroups of NK cells express activating Ly49 receptors with different specificities, is that rat NK cells can be induced into a memory state, i.e., selective expansion of the subset expressing the receptor for the immunizing ligand.

In a series of experiments conducted by Eva Petersson and coworkers BN rats (*RT1*^n^) immunized i.p. with cells expressing RT1 of a different MHC haplotype (*RT1*^u^) led to selective accumulation in the peritoneum of NK cells with specificity for target cells expressing a particular non-classical RT1-CE^u^ molecule and not classical *RT1.A*^u^ nor to third party MHC molecules. Conversely, immunization of BN rats with cells expressing RT1^c^ molecules led to expansion of anti RT1^c^ and not anti RT1^u^ specific NK cells. Long lasting immunity was not investigated in these experiments. However, these experiments showed clearly that alloreactive NK cells can be subdivided into subsets with activating receptors for different RT1 alleles. Furthermore, these subsets can be selectively expanded upon immunization with the cognate MHC ligand in the *RT1-CE* region akin to an adaptive immune response (71). Extended studies along this line has clearly demonstrated long lasting specific memory functions among mouse NK cells expressing activating Ly49 receptors for a virally encoded ligand (72) Such studies may have implications for vaccination strategies.

Whether the specialized function of the rat *RT1-CE* encoded MHC-I molecules in being ligands mainly for NK cell receptors is unique for the rat or similar regions exist in other species, remains to be shown. Nevertheless, the formation of a repertoire of activating Ly49 receptors can pave the path for inducing specific and possibly long lasting immunological memory among rat NK cells as it has been shown in the mouse (72).

## Rat NK Cells and Diseases

The rat serves as an animal model for several spontaneous or induced autoimmune diseases like Rheumatoid Arthritis (73, 74), diabetes (75, 76), autoimmune thyroiditis (77), and multiple sclerosis (experimental allergic encephalomyelitis, EAE) (78, 79). The rat strains where these disease states are present are different from those used by us: usually LEW, F344, WF, or BB. Their MHC and NKC with Ly49 genes are not yet fully characterized, so putative linkage between Ly49 receptor repertoire, expression of specific MHC-I molecules and disease is not yet achievable. A complete next generation sequencing of the NKC of these rat strains is needed to explore this field further. It is, however, noteworthy that our founder rat strain PVG, having a most extensive repertoire of both activating and inhibitory Ly49 receptor genes, shows no signs of either autoimmune diseases or spontaneous tumors.

Another issue pertaining to NK cells in disease is their putative role as therapeutic targets in allogeneic hematopoietic stem cell transplantation (HSCT) for the treatment of malignancies, especially leukemias. We have established a protocol where rats after myeloablative treatment (irradiation) are transplanted with MHC mismatched mononuclear BMCs from rat strains with full or partial mismatches for MHC genes, i.e., MHC-I or MHC-II/III genes. Host NK cells remaining after myeloablation are clearly important for survival or death of the transplanted HSC, and the mismatches that lead to failure of the transplanted BMC to engraft are identical to the mismatches where host NK cells are shown to kill hematopoietic cells of the donor by the ALC mechanism *in vivo* and *in vitro*. Depending on the haplotype differences between donor and recipient both classical *RT1-A* and non-classical *RT1-CE* MHC-I regions contribute (80, 81). The implication from these studies is that in a human alloHSCT setting the HSC donor and recipient should be chosen so that host alloreactive NK cells do not recognize MHC-mismatches on the transplanted hematopoietic cells which will lead to their destruction.

Alloreactivity of the donor-derived NK cells against host hematopoietic cells may, however, have a beneficial role. There is accumulating evidence that human patients treated for malignancies with alloHSCT have reduced relapse of the disease if the HSC are from a donor whose alloreactive NK cells can recognize MHC-I antigen mismatches in the recipient. This is particularly evident in the treatment of certain leukemias like AML (82). Initially, this was assigned to inhibitory KIR of the donor recognizing missing self MHC ligands in the recipient (82). However, more recent studies have shown that certain activating KIR like KIR2DS1 recognizing HLA-C2 may also be beneficial in that they also kill host antigen presenting cells and T cells and therefore may hinder the development of graft-versus-host disease (GVHD), the most serious complication seen after alloHCST (83–85). The beneficial role of alloreactive NK cells in both controlling GVH and having a protective effect against relapse of the leukemia (graft-versus-leukemia, GVL) has led to ongoing clinical trials where alloHSCT patients are given allogeneic NK cells as therapeutic tools for avoiding GVH and promoting GVL.

In our alloHSCT model in the rat, we can test the role of cellular therapy with alloreactive NK cells in preventing GVHD and in promoting GVL. We have tested the role of alloHCST in controlling an experimentally induced acute rat leukemia in the BN strain (BNML), resembling human AML (86, 87). Protection was obtained with repeated alloHSCT, but the mechanisms of protection are still elusive. Further research along these lines will be to extend these studies to encompass therapy with alloreactive NK cells, which are optimally cultured to promote the generation of a memory phenotype. These studies will be given high priority in our research group in the years to come.

## Conflict of Interest Statement

The author declares that the research was conducted in the absence of any commercial or financial relationships that could be construed as a potential conflict of interest.
